# Correlation of LMP10 Expression and Clinical Outcome in Human Papillomavirus (HPV) Positive and HPV-Negative Tonsillar and Base of Tongue Cancer

**DOI:** 10.1371/journal.pone.0095624

**Published:** 2014-04-21

**Authors:** Nikolaos Tertipis, Linnea Haeggblom, Cecilia Nordfors, Nathalie Grün, Anders Näsman, Andrea Vlastos, Tina Dalianis, Torbjörn Ramqvist

**Affiliations:** Department of Oncology-Pathology, Karolinska Institutet, Stockholm, Sweden; The Chinese University of Hong Kong, Hong Kong

## Abstract

**Aim:**

To examine LMP10 expression and its possible impact on clinical outcome in human papillomavirus (HPV) positive and HPV-negative tonsillar and base of tongue squamous cell carcinoma (TSCC and BOTSCC).

**Background:**

Outcome is better in HPV-positive TSCC and BOTSCC compared to matching HPV-negative tumours, with roughly 80% vs. 40% 5-year disease free survival (DFS) with less aggressive treatment than today’s chemoradiotherapy. Since current treatment often results in harmful side effects, less intensive therapy, with sustained patient survival would be an attractive alternative. However, other markers together with HPV status are necessary to select patients and for this purpose LMP10 expression is investigated here in parallel to HPV status and clinical outcome.

**Materials and Methods:**

From 385 patients diagnosed between 2000 and 2007 at the Karolinska University Hospital, 278 formalin fixed paraffin embedded TSCC and BOTSCC biopsies, with known HPV DNA status, were tested for LMP10 nuclear and cytoplasmic expression (fraction of positive cells and staining intensity). The data was then correlated to clinical outcome.

**Results:**

An absent/low compared to a moderate/high LMP10 nuclear fraction of positive cells was correlated to a better 3-year DFS in the HPV-positive group of patients (log-rank p = 0.005), but not in the HPV-negative group. In the HPV-negative group of patients, in contrast to the HPV-positive group, moderate/high LMP10 cytoplasmic fraction and weak/moderate/high LMP10 cytoplasmic intensity correlated to a better 3-year DFS (p = 0.003 and p = 0.001) and 3-year overall survival (p = 0.001 and 0.009).

**Conclusion:**

LMP10 nuclear expression in the HPV-positive group and LMP10 cytoplasmic expression in the HPV-negative group of patients correlated to better clinical outcome.

## Introduction

In 2007, the International Agency for Cancer Research (IARC) acknowledged human papillomavirus (HPV) as a risk factor for oropharyngeal squamous cell carcinoma (OPSCC) based on data by others and us [1], [2], [3], [4]. Moreover, HPV-positive OPSCC, with tonsillar and base of tongue squamous cell cancer (TSCC and BOTSCC) accounting for 80% of the cases, has a better prognosis compared to the corresponding HPV-negative tumours (80% vs. 40%–60% 3-year disease free survival (DFS)[1], [3], [4], [5], [6], [7], [8], [9], [10], [11], [12]. In parallel with a recent increase in the incidence of HPV-positive OSCC in many countries, curative therapy for head and neck squamous cell carcinoma (HNSCC), including OPSCC, has become more aggressive with e.g. chemo-radiotherapy resulting in more serious adverse effects [13], [14], [15], [16], [17], [18], [19], [20], [21], [22], [23], [24]. Less aggressive treatment would likely be sufficient for most patients with HPV-positive TSCC and BOTSCC, where HPV is most commonly found and where HPV has the greatest impact on survival [25]. Nevertheless, before tapering treatment, other markers in combination with HPV status would be of use to select patients with HPV-positive TSCC and BOTSCC with a very likely favourable response to therapy [26], [27], [28], [29], [30], [31], [32]. Recent studies have identified some markers, e.g. absent/low HLA class I or CD44 intensity expression, or high CD8+ tumour infiltrating lymphocyte (TIL) counts in HPV-positive TSCC and BOTSCC, which all shown to be favourable prognostics factors and indicate a 95–100% probability of 5-year disease specific survival [26], [27], [28], [29], [30], [31], [32]. Unfortunately, the above biomarkers only recognize a fraction of all patients with a good probability to respond well to treatment and for that additional biomarkers to the ones above are needed.

LMP10 (low molecular weight protein 10) is a beta subunit of the 20S proteasome, identified as a third IFN-g inducible unit by Nandi et al 2006 [33]. Together with LMP2 and LMP7, LMP10 build up the so-called immunoproteasome acting upon intensified immune response. As a component of the antigen processing machinery (APM), LMP10 is implicated in the pathway of peptide trimming during peptide presentation by HLA molecules. Defects in different APM components have been found in many tumours and have shown correlation with clinical outcome of patients [34], [35], [36], [37].

In this study, we therefore examined for LMP10 expression in HPV-positive and HPV-negative TSCC and BOTSCC, in 278 patients diagnosed between 2000–2007 at the Karolinska University Hospital, and any correlation of LMP10 expression to survival.

## Patients, Materials and Methods

### Ethics Statement

The study was performed according to ethical permissions 2005/431-31/4, 2005/1330-32 and 2009/1278-31/4 from the Regional Ethical Committee at Karolinska Institutet, Stockholm, Sweden. Written informed consent was obtained from all participants in the study.

### Patients and Biopsies

During 2000–2007, 385 patients diagnosed with TSCC and BOTSCC (defined as ICD-10 codes: C09, C01.9 respectively) at the Karolinska University Hospital, were identified through the Swedish Cancer Registry. Of these, 278 patients, with diagnostic pre-treatment biopsies, previously tested for HPV DNA and with sufficient material, were included in this study [27]. The 258 patients treated with curative intent were then comprised in the survival analysis. Conventional radiotherapy (RT) (2.0 Gy/day, for 6.5–7 weeks, total dose: 68 Gy) or accelerated RT (1.1+2.0 Gy/day for 4.5 weeks, total dose: 68 Gy) was given to 192/258 (74.4%) of the patients, while 66/258 (25.6%) patients also had induction chemotherapy and concomitant RT. Interstitial radiation (Brachytherapy) was also given to some patients (78 Gy totally). Patients with nodal disease underwent neck dissection 6–8 weeks after completed RT. Patients were followed every 3 months for the first 2 years, and every 6 months during the 3rd year, and their survival data were obtained from patients’ records. The study was performed according to ethical permissions 2005/431-31/4, 2005/1330-32 and 2009/1278-31/4 from the Regional Ethical Committee at Karolinska Institutet, Stockholm, Sweden.

### HPV Analysis

HPV DNA of 27 HPV types and betaglobin was assayed by a bead-based multiplex assay on a Magpix instrument (Luminex Corporation) as previously described [30].

### Immunohistochemistry

In brief, formalin fixed paraffin embedded (FFPE) tumour biopsy slides (4 µm) were de-paraffinized in xylene and rehydrated in ethanol of decreasing concentrations. Afterwards they were heated in citrate buffer (pH 6.0) for antigen retrieval. Unspecific sites were blocked with 1.5% horse serum diluted in phosphate buffered saline (PBS). Paraffin sections were incubated overnight at +8°C with primary antibody, MECL-1 C-2 (anti-LMP10 antibody from Santa Cruz Biotechnology), in a moist chamber followed by secondary antibody application and a standard ABC-HRP kit for antigen detection. The chromogen-3′-diaminobenzydine (DAB) was used for visualization. Haematoxylin was applied for counterstaining followed by dehydration in ethanol of increasing concentrations and xylene.

### Evaluation of Immunohistochemistry Staining

**Figure 1:**
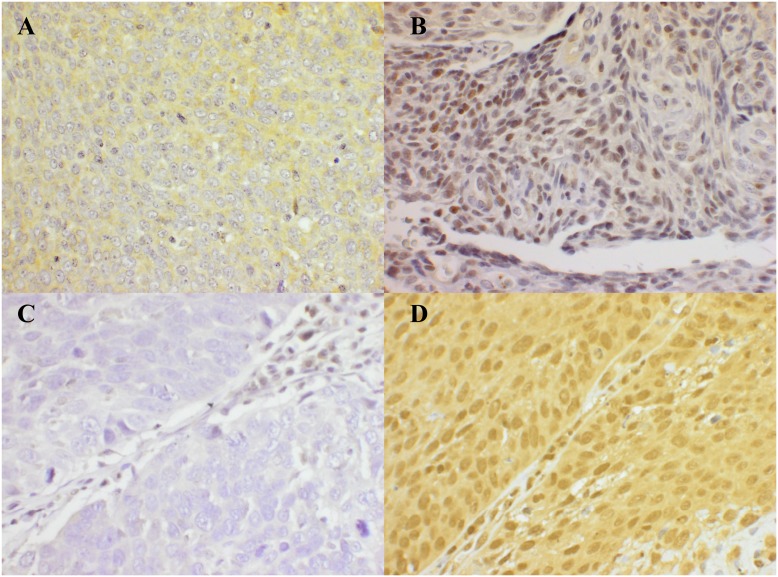
Example of LMP10 nuclear and cytoplasmic expression in TSCC/BOTSCC. (A): Weak/moderate cytoplasmic expression of LMP10; (B): Moderate/high nuclear and absent cytoplasmic expression of LMP10; (C): Absent or undetectable LMP10 nuclear and cytoplasmic expression; (D): Moderate nuclear and cytoplasmic expression of LMP10. Magnification 400X.

LMP10 cytoplasmic and nuclear staining, including negative and positive controls (Figure 1), was evaluated by two independent researchers blinded for data and clinical outcome, similar to that described previously [32]. The fraction of malignant cells stained for LMP10 in the nucleus and the cytoplasm was evaluated separately semi-quantitatively in 4 grades of percentages of stained tumour cells: 0 (0%), 1 (1–25%), 2 (26–75%) or 3 (76–100%). The intensity of the staining was scored separately and evaluated as absent, weak, moderate, and strong staining. The intensity of staining was defined by comparing the intensity of the staining in the malignant cells, with the staining of internal controls (normal lymphoid tissue) per batch. In the minority of cases where the staining within the same sample was not even, the intensity of the majority of the cells was used in the analysis. Staining and evaluation of HLA class I antigens were performed on the same tumour samples in two earlier studies as described elsewhere [27], [28].

### Statistical Analysis

Both patient characteristics and categorical data were analysed using the Chi square-test or, for comparison of mean values, the student T-test. Two-tailed p-values (5% significance level) were reported for all analyses. Survival was measured in days of diagnosis until an event occurred, or until 3 years after diagnosis when patients were censored. Death due to any cause (event) was included in the definition of overall survival (OS), while only disease recurrence (TSCC or BOTSCC) was used for calculation of disease free survival (DFS). Notably, death of patients without any prior recurrence was censored at day 0 when calculating DFS. Kaplan-Meier estimator was used for DFS and OS, and differences in the survival of patients were tested using the log-rank test. The Cox proportional hazards model was used for the calculation of the adjusted and unadjusted hazard ratios (HRs). All calculations and analyses were performed using IBM Corp., SPSS Statistics, version 21.0.

## Results

### Patients, Tumour Characteristics and HPV

The characteristics of the 278 patients, 195 (70%) with TSCC and 83 (30%) with BOTSCC and their tumours are shown in Table 1. In total 207/278 (74%) of the tumours were HPV-positive. Patients with HPV-positive tumours were younger (mean and median age 60) than those with HPV-negative tumours (mean and median age 64 and 62 years respectively) (p<0.001) (Table 1, and data not shown). In total 258/278 (92.8%) patients received treatment with curative intent (Table 1), 199/207 (96%) of those with HPV-positive tumours (median age of 60 years), and 59/71 (83%) of those with HPV-negative tumours (median age 62 years). Only the 258 patients treated with curative intent were included in the analysis of 3-year DFS and OS.

**Table 1:** Characteristics of patients with TSCC and BOTSCC and their tumours.

	HPV-positive tumours		HPV-negative tumours		All patients/tumours		p-value*
Patient Characteristics	207		71		278		
	N	%	N	%	N	%	
**Age (years)**							
Mean	60		64		61		**<0.001**
Median	60		62		61		
Range	30–90		44–100		30–100		
**Diagnosis**							
TSCC	144	69.6%	51	71.8%	195	70.1%	0.72
BOTSCC	63	30.4%	20	28.2%	83	29.9%	
**Sex**							
Male	156	75.4%	54	76.1%	210	75.5%	0.84
Female	51	24.6%	17	23.9%	68	24.5%	
**Tumor size**							
T1	52	25.1%	12	16.9%	64	23.0%	**0.018**
T2	74	35.7%	16	22.5%	90	32.4%	
T3	36	17.4%	21	29.6%	57	20.5%	
T4	45	21.7%	22	31.0%	67	24.1%	
**Nodal disease**							
N0	27	13.0%	29	40.8%	56	20.1%	**<0.001**
N1	50	24.2%	9	12.7%	59	21.2%	
N2a	36	17.4%	5	7.0%	41	14.7%	
N2b	63	30.4%	11	15.5%	74	26.6%	
N2c	22	10.6%	10	14.1%	32	11.5%	
N3	9	4.3%	7	9.9%	16	5.8%	
NX	0	0.0%	0	0.0%	0	0.0%	
**Distant metastasis**							
M0	200	96.6%	69	97.2%	269	96.8%	0.84
M1	5	2.4%	1	1.4%	6	2.2%	
MX	2	1.0%	1	1.4%	3	1.1%	
**Stage**							
I	1	0.5%	9	12.7%	10	3.6%	**<0.001**
II	12	5.8%	5	7.0%	17	6.1%	
III	53	25.6%	17	23.9%	70	25.2%	
IVa	126	60.9%	31	43.7%	157	56.5%	
IVb	10	4.8%	9	12.7%	19	6.8%	
Ivc	5	2.4%	0	0.0%	5	1.8%	
**Treatment**							
Curative	199	96.1%	59	83.1%	258	92.8%	**<0.001**
Palliative	8	3.9%	12	16.9%	20	7.2%	
**Smoking**							
Never	67	32.4%	5	7.0%	72	25.9%	**<0.001**
Former (>15years ago)	38	18.4%	3	4.2%	41	14.7%	
Former (<15years ago)	38	18.4%	4	5.6%	42	15.1%	
Current at diagnosis	58	28.0%	50	70.4%	108	38.8%	

*p-value for comparison of HPV-positive *vs*. HPV-negative tumours/patients.

### LMP10 Expression and HPV in TSCC and BOTSCC

LMP10 nuclear and cytoplasmic expression was evaluated for all 278 patients and is exemplified in Figure 1. LMP10 nuclear expression did not show any significant difference between the TSCC and BOTSCC groups, neither with regard to the fraction of positive cells nor with the intensity of staining (p = 0.115 and p = 0.313, respectively, Table 2). However, the fraction of LMP10 cytoplasmic positive cells was relatively higher in the HPV-positive as compared to the HPV-negative group (p = 0.033), while there was no significant difference in LMP10 cytoplasmic intensity staining between the groups (p = 0.211, Table 2).

**Table 2:** LMP10 expression in HPV-positive and HPV-negative TSCC and BOTSCC.

		HPV-positive		HPV-negative		All tumours		p value§
		n = 207		n = 71		n = 278		
		n*	%	n	%	n	%	
Nucleus	Fraction							
	0%	83	40.1%	20	28.2%	103	37.1%	0.115
	1–25%	32	15.5%	13	18.3%	45	16.2%	
	26–50%	18	8.7%	4	5.6%	22	7.9%	
	51–75%	18	8.7%	13	18.3%	31	11.2%	
	76–100%	56	27.1%	21	29.6%	77	27.7%	
Nucleus	Intensity							
	Absent	83	40.1%	20	28.2%	103	37.1%	0.313
	Weak	46	22.2%	18	25.4%	64	23.0%	
	Moderate	71	34.3%	29	40.8%	100	36.0%	
	Strong	7	3.4%	4	5.6%	11	4.0%	
Cytoplasm	Fraction							
	0%	82	39.6%	35	49.3%	117	42.1%	0.033
	1–25%	26	12.6%	14	19.7%	40	14.4%	
	26–50%	22	10.6%	10	14.1%	32	11.5%	
	51–75%	29	14.0%	4	5.6%	33	11.9%	
	76–100%	48	23.2%	8	11.3%	56	20.1%	
Cytoplasm	Intensity							
	Absent	82	39.6%	35	49.3%	117	42.1%	0.211
	Weak	100	48.3%	33	46.5%	133	47.8%	
	Moderate	24	11.6%	3	4.2%	27	9.7%	
	Strong	1	0.5%	0	0.0%	1	0.4%	

§ Chi-square test.

*n denotes the number of tumours.

### LMP10 Nuclear Staining and HPV Status in Correlation to Clinical Outcome

**Figure 2:**
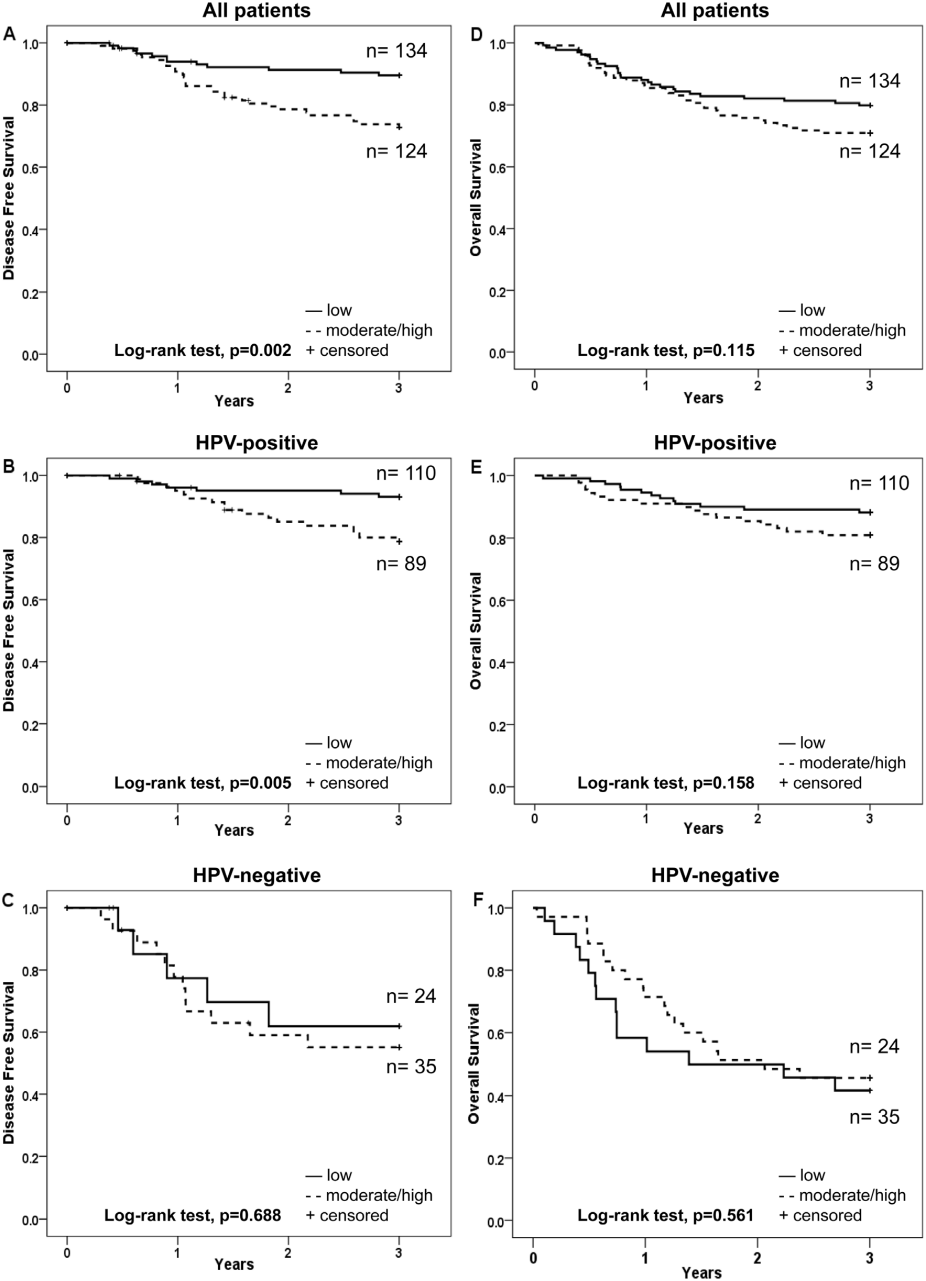
Kaplan-Meier curves for disease free survival (DFS) and overall survival (OS) of patients with TSCC or BOTSCC, treated with intention to cure and stratified according to fraction of cells positive for nuclear expression of LMP10. A and D: DFS (A) and OS (D) for TSCC and BOTSCC irrespective of the HPV status of the tumour. B and E: DFS (B) and OS (E) of patients with HPV-positive TSCC and BOTSCC. C and F: DFS (C) and OS (F) of patients with HPV-negative TSCC and BOTSCC.

**Figure 3:**
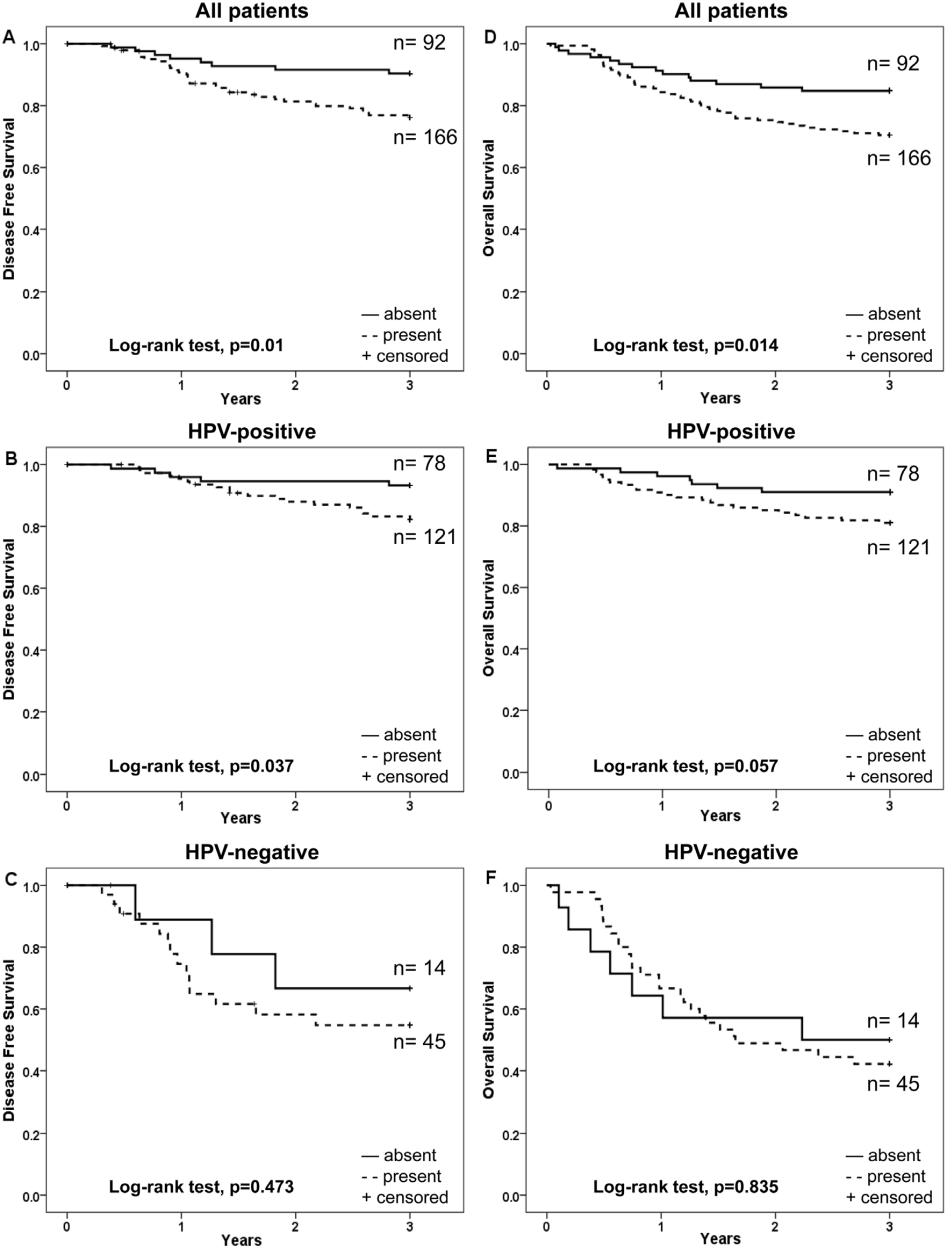
Kaplan-Meier curves for disease free survival (DFS) and overall survival (OS) of patients with TSCC or BOTSCC, treated with intention to cure and stratified according to intensity of nuclear expression of LMP10. A and D: DFS (A) and OS (D) for TSCC and BOTSCC irrespective of the HPV status of the tumour. B and E: DFS (B) and OS (E) of patients with HPV-positive TSCC and BOTSCC. C and F: DFS (C) and OS (F) of patients with HPV-negative TSCC and BOTSCC.

LMP10 nuclear expression was evaluated both for the fraction of positive cells in the nucleus, and for the intensity of nuclear staining, and was correlated to survival of the 258 patients treated with curative intent (illustrated as Kaplan-Meier curves in Figures 2 and 3 respectively).

An absent/low (≤25%), as compared to a moderate/high (26–100%), fraction of LMP10 nuclear positive cells correlated to a better 3-year DFS in the whole group of patients, and for patients with HPV-positive tumours, but not among patients with HPV-negative tumours (log-rank test, p = 0.002, p = 0.005 and p = 0.688 respectively) (Figure 2 A–C). There was however no significant difference in 3-year OS, when comparing similar parameters, for the whole group, the HPV-positive and the HPV-negative groups (log-rank test, p = 0.115, p = 0.158 and 0.561 respectively) (Figure 2 D–F).

An absent, as compared to weak/moderate/strong (present), LMP10 nuclear intensity staining was correlated with a better 3-year DFS for the whole group (p = 0.01) and the HPV-positive group (p = 0.037), but not for the HPV-negative group (p = 0.473) (Figure 3A–C). For 3-year OS this was only observed for the whole group of patients (p = 0.014) (Figure 3 D).

The possible influence of LMP10 nuclear expression as well as LMP10 nuclear intensity staining was pursued separately for the HPV-positive and HPV-negative groups.

A multivariate Cox-regression analysis was performed for patients with HPV-positive and HPV-negative tumours separately, including the fraction of LMP10 nuclear positive cells or LMP10 nuclear intensity, age and tumour stage, with regard to 3-year DFS and 3-year OS. For the HPV-positive group an absent/low fraction as compared to a moderate/high fraction of LMP10 positive cells was still a determinant of a better 3-year DFS (p = 0.027) (Table 3), but not age or stage, (p = 0.079 and p = 0.744) (data not shown). Furthermore, none of these parameters were determinants of 3-year OS, except for age (p = 0.006, data not shown) (Table 3). LMP10 nuclear intensity staining did not reach statistical significance for either 3-year DFS or 3-year OS (Table 3). The corresponding analysis for the HPV-negative group suggested that nuclear LMP10 expression had no impact on clinical outcome in this group (3-year DFS and 3-year OS) (Table 3).

**Table 3:** Univariable and multivariable analyses on LMP10 staining of TSCC and BOTSCC in relation to DFS and OS**.**

		DFS						OS					
		Univariable			Multivariable§			Univariable			Multivariable§		
		HR	95% CI	p-value	HR	95.0% CI	p-value	HR	95.0% CI	p-value	HR	95.0% CI	p-value
HPV-positive													
Nucleus	Fraction*	3.254	1.349–7.850	**.009**	2.755	1.121–6.767	**.027**	1.673	0.813–3.445	.162	1.340	0.640–2.805	.438
	Intensity**	2.729	1.019–7.310	**.046**	2.253	0.827–6.135	.112	2.228	0.956–5.194	.063	1.792	0.754–4.261	.187
Cytoplasm	Fraction	2.070	0.886–4.838	.093	1.869	0.797–4.382	.150	.590	0.281–1.240	.164	.521	0.247–1.098	.086
	Intensity	1.494	0.620–3.604	.371	1.346	0.557–3.254	.509	.703	0.343–1.441	.336	.617	0.300–1.269	.189
HPV-negative													
Nucleus	Fraction	1.238	0.436–3.518	.688	1.274	0.447–3.631	.650	.815	0.408–1.627	.562	.958	0.474–1.935	.905
	Intensity	1.573	0.452–5.481	.477	1.645	0.461–5.871	.443	1.092	0.474–2.519	.836	1.194	0.516–2.764	.679
Cytoplasm	Fraction	.143	0.033–0.631	**.010**	.154	0.035–0.684	**.014**	.234	0.090–0.610	**.003**	.281	0.107–0.739	**.010**
	Intensity	.204	0.071–0.584	**.003**	.219	0.073–0.653	**.006**	.405	0.201–0.818	**.012**	.487	0.236–1.004	.051

§ Adjusted for age and tumor stage,

*absent/low vs moderate/high,

**absent vs weak/moderate/high.

Abbreviations: HR, Hazard Ratio; CI, Confidense Interval.

### LMP10 Cytoplasmic Staining and HPV Status in Correlation to Clinical Outcome

**Figure 4:**
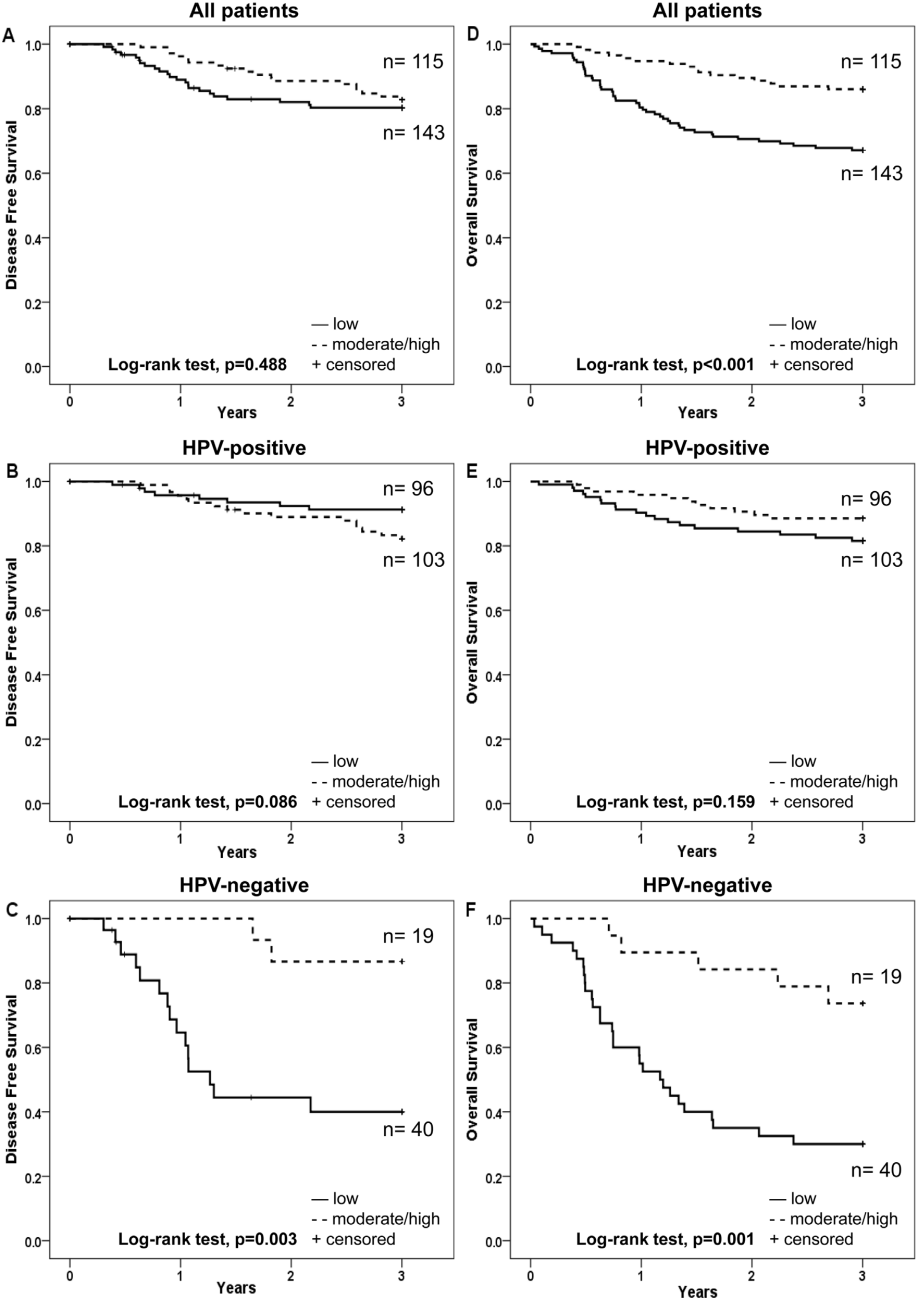
Kaplan-Meier curves for disease free survival (DFS) and overall survival (OS) of patients with TSCC or BOTSCC, treated with intention to cure and stratified according to fraction of cells positive for cytoplasmic expression of LMP10. A and D: DFS (A) and OS (D) for TSCC and BOTSCC irrespective of the HPV status of the tumour. B and E: DFS (B) and OS (E) of patients with HPV-positive TSCC and BOTSCC. C and F: DFS (C) and OS (F) of patients with HPV-negative TSCC and BOTSCC.

**Figure 5:**
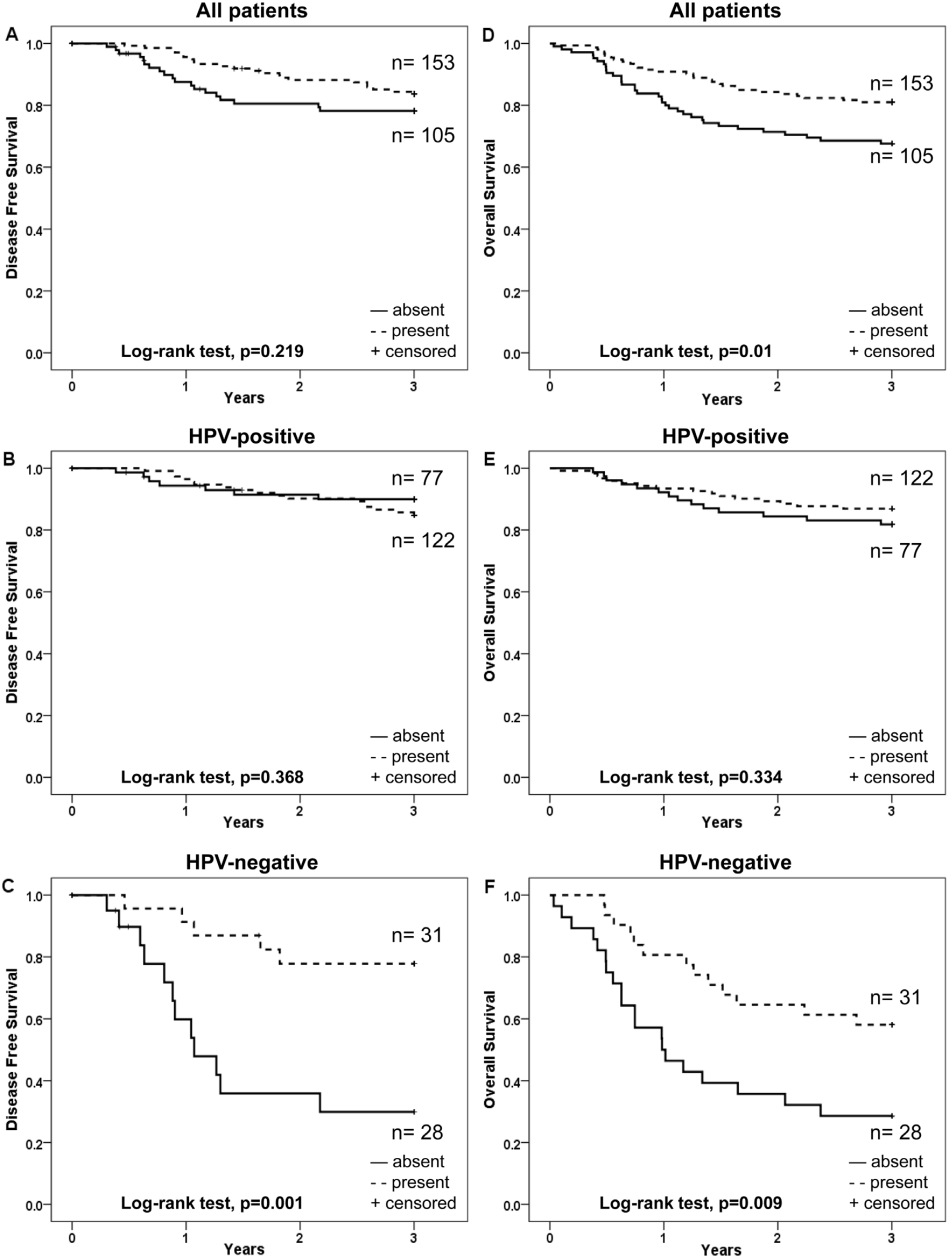
Kaplan-Meier curves for disease free survival (DFS) and overall survival (OS) of patients with TSCC or BOTSCC, treated with intention to cure and stratified according to intensity of cytoplasmic expression of LMP10. A and D: DFS (A) and OS (D) for TSCC and BOTSCC irrespective of the HPV status of the tumour. B and E: DFS (B) and OS (E) of patients with HPV-positive TSCC and BOTSCC. C and F: DFS (C) and OS (F) of patients with HPV-negative TSCC and BOTSCC.

LMP10 cytoplasmic expression was evaluated both for the fraction of positive cells and intensity of staining and was correlated to survival for the 258 patients treated with curative intent (illustrated as Kaplan-Meier curves in Figures 4 and 5 respectively).

A moderate/high as compared to an absent/low fraction of LMP10 cytoplasmic positive cells correlated to a better 3-year DFS for patients with HPV-negative tumours but not for the whole group of patients, nor for the patients with HPV-positive tumours, (log-rank test, p = 0.003, p = 0.488 and p = 0.086, respectively (Figure 4 A–C). For 3-year OS the corresponding correlation was significant both for the HPV-negative and the whole group, but not for the HPV-positive group (log-rank test, p = 0.001, p<0.001 and p = 0.159, respectively), (Figure 4 D–F).

Weak/moderate/high (present) LMP10 cytoplasmic intensity staining as compared to absent LMP10 staining was correlated to a better 3-year DFS for patients with HPV-negative tumours, but not for the whole group or for patients with HPV-positive tumours (log-rank test p = 0.001, p = 0.219, and 0.368, respectively) (Figure 5 A–C). For 3-year OS the corresponding correlation was significant both for the HPV-negative and the whole group, but not for the HPV-positive group (log-rank test p = 0.009, p = 0.01 and p = 0.334, respectively (Figure 5 D–F).

The possible influence of LMP10 cytoplasmic expression was pursued separately for the HPV-positive and HPV-negative groups (Table 3). For that a multivariate Cox-regression analysis was performed for patients with HPV-positive and HPV-negative tumours, including the fraction of LMP10 cytoplasmic positive cells, age and stage, with regard to 3-year DFS and 3-year OS. A corresponding analysis was performed for LMP10 cytoplasmic intensity. For patients with HPV-negative tumours, both a moderate/high fraction of LMP10 cytoplasmic positive cells, and the presence of LMP10 cytoplasmic staining was correlated to a better 3-year DFS (p = 0.014 and p = 0.006, respectively) (Table 3). The corresponding figures for 3-year OS were p = 0.010 and p = 0.051 respectively (Table 3). Age and stage did not reach statistical significance in the analysis for patients with HPV-negative tumours (data not shown).

The corresponding analysis for the HPV-positive group suggested that cytoplasmic LMP10 expression had no impact on clinical outcome (3-year DFS and 3-year OS) (Table 3).

### LMP10 Expression, HLA Class I Expression and HPV Status in Correlation to Clinical Outcome

In recent studies we have demonstrated that absent or low HLA class I expression was correlated to better survival in patients with HPV-positive TSCC and BOTSCC, while the opposite was the case for patients with HPV-negative TSCC and BOTSCC [27], [28]. To investigate if the correlation of LMP10 expression predicts survival independent of HLA class I expression or not, two multivariate Cox-regression analyses with regard to 3-year DFS were performed, with HLA class I data obtained from Näsman et al [27].

In the first analysis, HPV-positive TSCC and BOTSCC were examined and the analysis included the fraction of LMP10 nuclear positive cells, age, stage and HLA class I expression assayed as intensity of HCA2-staining. As presented in Table 4, both LMP10 and HCA2 intensity were independent predictors of 3-year DFS.

**Table 4:** Multivariable analysis of LMP10 and HLA class I expression on TSCC/BOTSCC in relation to DFS.

DFS							
HPV-positive				HPV-negative			
Variables	HR	95% CI	p-value	Variables	HR	95% CI	p-value
LMP10 fraction nucleus*	2.817	1.145–6.928	**.024**	LMP10 fraction cytoplasm*	.124	0.027–0.565	**.007**
HCA2 intensity**	3.326	1.369–8.076	**.008**	HCA2 fraction*	.267	0.092–0.775	**.015**
Age	1.035	0.993–1.079	.105	Age	.995	0.940–1.054	.870
Stage I-IIIvsIV	1.017	0.442–2.340	.969	Stage I-IIIvsIV	1.184	0.447–3.136	.734

*absent/low vs moderate/high,

**absent/weak vs strong.

Abbreviations: HR, Hazard Ration; CI, Confidence Interval.

In the second analysis, HPV-negative TSCC and BOTSCC were studied and the analysis contained the fraction of LMP10 cytoplasmic positive cells, age, stage and the fraction of HLA class I positive cells assayed by HCA2-staining. As presented in Table 4, both the fraction of LMP10 and HCA2 positive cells were independent predictors of 3-year DFS.

## Discussion

The present study investigated LMP10 expression both in the nuclear and cytoplasmic compartments, in relation to HPV status and clinical outcome in 195 TSCC and 83 BOTSCC cases. LMP10’s nuclear and cytoplasmic expression, including both the fraction of positive cells and the intensity of staining was similar in HPV-positive and HPV-negative tumours, except for the higher fraction of LMP10 cytoplasmic positive cells in the HPV-positive group.

Furthermore, an absent/low fraction of LMP10 positive cells in the nucleus, as well as absent nuclear staining intensity correlated to a better 3-year DFS for patients with HPV-positive tumours, but not for those with HPV-negative tumours. The reverse was found for cytoplasmic LMP10 staining, where a moderate/high fraction of positive cells and weak/moderate/high intensity was correlated to better 3-year DFS and OS in the HPV-negative, but not in the HPV-positive group.

The reason for the difference with regards to nuclear and cytoplasmic LMP10 staining and survival for the HPV-positive and HPV-negative groups is not obvious. In earlier studies LMP10 cytoplasmic expression has been evaluated in bladder and oesophageal cancer for both the fraction of positive cells and the intensity of staining, but was not shown to have any prognostic impact [34], [35], [37]. However, in these studies there was no separate analysis of nuclear and cytoplasmic expression. In this study, evaluation and analysis of nuclear and cytoplasmic staining provided additional information.

To our knowledge there are no prior studies on LMP10 expression in relation to survival in HNSCC. Meissner *et al.* demonstrated a correlation with survival in HNSCC for both LMP2 and LMP7 [38] and in a recent study, expression of LMP7 was also correlated to survival in salivary cancer [39]. However, the tumours were not analysed for the presence of HPV in any of these studies.

The finding that a low nuclear LMP10 expression correlated to a better 3-year DFS in the HPV-positive group, while a corresponding high cytoplasmic LMP10 expression correlated to a better 3-year DFS in the HPV-negative group suggests several implications. First of all, our study suggests that LMP10 could be used as a prognostic marker for patients with either HPV-positive or HPV-negative tumours. We have previously been able to show that a biomarker, in the same pathway, HLA class I expression, can be used in a differential way to predict clinical outcome in HPV-positive and HPV-negative tumours. Thus, an absent/low HLA class I expression is a positive prognostic factor for patients with HPV-positive tumours, while the opposite is true for patients with HPV-negative tumours, where a high HLA class I expression is of benefit [27]. Thus, both with regard to HLA class I and LMP10 expression in relation to survival, there were differences between HPV-positive and HPV-negative tumours, although these were more pronounced for HLA class I. Notably, as demonstrated by the multivariate analysis in Table 4, expression of LMP10 and HLA class I were independent predictors of survival for both HPV-positive and HPV-negative tumours. The underlying mechanisms behind the regulation of LMP10 and HLA class I expression in these tumours should thus at least in part be separate.

It is not known if the presence of HPV can influence LMP10 expression directly, and even if this is the case, the mechanism behind this regulation is not known. It has been shown that HPV E5 has the potential to down-regulate the expression of HLA class I [40]. Whether E5 also affects the expression of LMP10 has, to our knowledge, not been investigated. It has been shown that human papillomaviruses have the ability to regulate the HLA class I heavy chain as well as some of the antigen processing machinery components in the transcriptional level [41]. However, the transcriptional regulation of e.g. the LMP2 promoter was found to be dependent on the E7 protein of HPV18 and the low risk HPV 6b but not by E7 of HPV16. This suggests that there is a potential difference in the cellular effects caused by the E7 protein of HPV16 and HPV18. Noteworthy, the majority of the TSCC and BOTSCC cases are positive for HPV16. Whether expression of LMP10 can be regulated by E7 has, to our knowledge, not been investigated. Noteworthy, in the present study the fraction of LMP10 cytoplasmic positive cells was higher in the HPV-positive as compared to the HPV-negative group. However, there was no significant difference in LMP10 cytoplasmic intensity staining between the groups. This indicates the possibility of some but not a strong regulation by HPV in these tumour cells.

There are some limitations in our study. Primarily the study is retrospective, but this may not affect the results. In addition, there were fewer HPV-negative cases, which is a direct representation of the high HPV prevalence in OSCC in patients from Sweden today.

In conclusion, this study shows that LMP10 expression in TSCC and BOTSCC is correlated to disease free survival for both patients with HPV-positive and HPV-negative tumours and has the potential to be used in a clinical setting. However, since nuclear LMP10 expression predicts survival for patients with HPV-positive tumours, while cytoplasmic LMP10 expression correlates to survival for HPV-negative tumours, LMP10 expression would need to be analysed in relation to tumour HPV status.
